# Gendered speech development in early childhood: Evidence from a longitudinal study of vowel and consonant acoustics

**DOI:** 10.1017/S030500092500011X

**Published:** 2026-07

**Authors:** Eugene Wong, Kiana Koeppe, Margaret Cychosz, Benjamin Munson

**Affiliations:** 1 Department of Speech-Language-Hearing Sciences, University of Minnesota, Minneapolis, USA; 2 Department of Linguistics, University of California, Los Angeles, USA

**Keywords:** gender, phonological development, sociolinguistics

## Abstract

Adults rate the speech of children assigned male at birth (AMAB) and assigned female at birth (AFAB) as young as 2.5 years of age differently on a scale of *definitely a boy* to *definitely a girl* (Munson et al., 2022), despite the lack of consistent sex dimorphism in children’s speech production mechanisms. This study used longitudinal data to examine the acoustic differences between AMAB and AFAB children and the association between the acoustic measures and perceived gender ratings of children’s speech. We found differences between AMAB and AFAB children in two acoustic parameters that mark gender in adult speech: the spectral centroid of /s/ and the overall scaling of resonant frequencies in vowels. These results demonstrate that children as young as 3 years old speak in ways that reflect their sex assigned at birth. We interpret this as evidence that children manipulate their speech apparatus volitionally to mark gender through speech.

## 1. Introduction

A fundamental characteristic of human language is the ability to convey multiple types of information simultaneously. A single utterance of the word /kæt/ conveys linguistic meanings, including the specific meaning that should be invoked (i.e., the animal *Felis catus* or the name *Kat*), and the speaker’s broad intention for how their interlocutors should respond, i.e., whether it is a question or a statement of new information. The utterance also signals the *kind* of person who produced it. A person’s gender is one of the most vivid, immediate, and robust pieces of *person-kind* information that language conveys (Tripp & Munson, 2022). Gender differences in speech are so robust that listeners can perceive gender from short segments of individual speech sounds.

Gender is not identical to sex assigned at birth (SAB). SAB refers to determinations of whether someone is male, female, or intersex at birth, based largely on external inspections of gross morphology. Gender refers to one’s deeply felt identity as male, female, or a gender that is neither exclusively male nor exclusively female. The behaviours that individuals use to express their gender are culturally and socially specific and are not the inevitable consequence of a particular SAB. As reviewed by Munson and Babel (2019), gender differences in speech represent culturally and linguistically specific behaviours. Crucially, these differences go far beyond what would be expected solely from anatomical differences between adult cisgender men and women – i.e., men and women whose gender identities correspond to the sexes they were assigned at birth. Johnson (2006) showed that the magnitude of male–female differences in vowel formant frequencies varies considerably across different languages. These differences could not be explained by differences in body size (which correlates with a vocal-tract size, which in turn affects absolute formant frequencies) across the communities that speak those languages. The cultural and linguistic specificity of gendered speech implies that these behaviours must be learned. Given the many types of information that language conveys, children as language learners must acquire each of these types of information and how they interact with one another. The current investigation examines how children learn ways of speaking that convey their nascent gender, at least for cisgender children whose gender reflects cultural expectations that children assigned male at birth (AMAB) will develop male identities and children assigned female at birth (AFAB) will develop female identities.

The development of gender identity in childhood is a multifaceted and protracted process (Perry et al., 2019). The majority of children categorize themselves according to cisnormative expectations by 3 years of age. AMAB children categorize themselves as male, and AFAB children categorize themselves as female (Martin & Ruble, 2004). By about 5 years of age, some trans children begin to categorize themselves as being a gender that does not meet cisnormative expectations (Olson et al., 2015).

In addition to developing their own gender identity, preschool children also develop awareness of how gender is reflected in the behaviours of adults and their peers, including how speech indexes gender. There is some evidence that children can perceive gender through speech cues even very early in life: by 6 months, infants are able to distinguish between adult men’s and women’s voices (Miller, 1983). By 8 months, infants show knowledge of (mis-)matches between male and female faces, and voices that are cisheteronormatively male or female (Patterson & Werker, 2002).

Knowledge of gender *stereotypes* also emerges early in life: children’s stereotyped knowledge of gender is evident in their knowledge of associations between maleness/femaleness and physical appearances, toys, activities, and occupations. For example, 18-month-olds associate male faces with fire hats, hammers, and bears (Eichstedt et al., 2002). By 3.5 years of age, children are aware that men have more access to resources and power to make decisions (Mandalaywala et al., 2020). Furthermore, knowledge of gender stereotypes and gendered differences in access to resources influences children’s behaviour. AFAB children prefer female-stereotyped toys – those that relate to household or nurturing activities, like dolls or dress-up games; while AMAB children prefer male-stereotyped toys – those related to danger and propulsion, like guns and trucks (Alexander et al., 2009; Ruble et al., 2006).

### 1.1. Gender differences in adults’ speech reflect learning

The speech of adult cisgender men and women differs along several phonetic dimensions. As a group, cisgender women have smaller, less-massive vocal folds (Titze, 1989; Whiteside, 1996) and shorter vocal tracts (Fitch & Giedd, 1999; Peterson & Barney, 1952) than cisgender men. This should predispose cis women to speak with a higher fundamental frequency (f0, perceived as a higher pitch) and higher formant frequencies than cisgender men, respectively. Indeed, adult women’s speech typically exhibits higher f0 and higher average formant frequencies than adult men’s speech (Hillenbrand et al., 1995). While there are absolute upper and lower bounds to an individual’s f0 and formant frequency ranges, individuals can volitionally manipulate f0 and average formant frequencies through different articulatory manoeuvres within the vocal tract, such as raising or lowering their larynx, protracting or protruding their lips, or increasing the tension of their vocal folds, which stretches and relaxes them (Ohala, 1984; Xu & Chuenwattanapranithi, 2007; Zhang, 2016). These articulatory gestures may result in someone’s voice being rated as more stereotypically masculine- or feminine-sounding. This active process likely explains why there is an imperfect correlation between direct measures of vocal-tract length (VTL) at rest and formant frequencies (Lammert & Narayanan, 2015).

There is ample experimental evidence that adults manipulate f0 and formant frequencies to express gender, including the findings of Johnson (2006). We interpret these findings as evidence of the use of phonetic variation to construct and express their gender. This can be seen in the construction of an artificial gender in performances: Cartei and Reby (2012) investigated how male actors acoustically manipulate their voices when performing gay characters. They found that male actors make articulatory manoeuvres that raise their f0 and formant frequencies, approximating some cis female speech norms. In a subsequent study, Cartei et al. (2012) showed that cisgender men and women also manipulate their f0 and formant frequencies when performing a more masculine or a more feminine persona. Cartei et al.’s studies indicate that people internalize gender stereotypes about the acoustic signatures of voices and actively modify these acoustic variables to convey gender in intentional performances.

The assertion that phonetic differences between men and women are both *learned* and *volitional* is consistent with empirical work and theoretical accounts of gender and language more broadly, as summarized by Zimman (2017), Eckert and Podesva (2021), Tripp and Munson (2022), among others. Sociolinguistic studies of gender and language argue that male–female differences, and the expression of gender more broadly, are evidence of *social agency* in language production. People select language forms to convey social meaning – identities and stances – to different audiences. One of the identities that people can convey through speech is gender. This is consistent with the work of Judith Butler (Butler, 2002). One key insight of Butler’s work is that the expression and construction of gender need not be to convey masculinity and femininity *per se*, but instead convey constellations of features that are associated on a societal level with maleness and femaleness. For example, consider speech clarity. Studies of English speakers have shown that women, as a group, produce clearer speech than men (Bradlow et al., 1996; Byrd, 1994; Munson et al., 2006). Moreover, people perceive clear speech to sound more feminine (Munson, 2007; Munson et al., 2006). The route between “clear” and “feminine” is not inevitable and instead reflects a combination of linguistic experiences, like hearing clear speech more frequently from women than from men, and ideologies, like believing that women *should* speak more clearly to meet societal expectations of gender roles (Holmes, 1997).

### 1.2. Male–female differences in /s/ reflect the learned expression of gender

The learned expression of gender through speech is illustrated by research on the widely studied case of variation in /s/ production. Jongman et al. (2000) reported a higher peak frequency in /s/ produced by women than men. They also found that /s/ produced by women had lower variance, or the distribution of spectral energy around the peak frequency, than /s/ produced by men. Tokens of /s/ with high spectral variance resemble the spectra of /θ/, can be misperceived as /θ/ and hence are characterized as “inaccurate” or “lisped.” Conversely, tokens with especially low spectral variance are perceived to be highly accurate and “sharp” (Calder, 2019; Holliday et al., 2015; Munson & Urberg Carlson, 2016). As a group, cisgender women have smaller vocal tracts than cisgender men, and smaller-sized cavities have higher resonant frequencies. In principle, it is possible that a smaller oral cavity downstream from the constriction of /s/ in women may have led to a higher peak frequency /s/ even in cases where the place of articulation was the same. The work of Fuchs and Toda (2010) refutes this hypothesis. Fuchs and Toda examined the relationships between palate size and /s/ acoustics in German and English speakers. Palate size reflects the size of the resonant cavity anterior to the fricative constriction. If /s/ differences between men and women were entirely due to vocal-tract size differences, then palate size should account for differences in men’s and women’s /s/. Fuchs and Toda found that, in both languages, women consistently produced a more fronted /s/ than men, and such a sex distinction in /s/ remains even after accounting for palate size differences between the speakers. It follows that the gender variation in /s/ is at least partially learned behaviour within social groups, rather than determined anatomically.

Fuchs and Toda’s conclusion is supported by studies that have also found that differences in /s/ between men and women are mitigated by other social variables like social class, age, and racial identity. Stuart-Smith (2007) examined the /s/ production of men and women from various groups of Glaswegians that varied in age and social class. Overall, the women produced /s/ with a higher mean peak frequency than the men, consistent with Jongman et al. However, younger, working-class women were found to produce a /s/ with a lower mean peak frequency than the younger middle-class women, the net result of which was a smaller difference in /s/ production between younger working-class men and women. There are clearly no differences in vocal-tract anatomy by social class; hence, these differences must reflect learned ways of speaking. Calder and King (2020) examined differences in spectral mean frequency between men’s and women’s /s/ in two Black communities in the US. They found no sex differences in one community (Bakersfield, CA) but robust differences in another (Rochester, NY). The authors argued that male–female differences in /s/ are specific to speech communities and sensitive to population structure. The variation in /s/ between men and women of different communities studied by Stuart-Smith and by Calder and King shows that /s/ differences between men and women are not the inevitable consequence of being male or female, but instead represent individuals marking locally relevant gendered identities through phonetic variation.

Further evidence of the learned expression of gender in speech comes from Zimman (2017), who investigated the interplay of f0 and /s/ variation in transmasculine adults. The individuals in Zimman’s study were taking testosterone to thicken their vocal folds and hence lower their f0. The length of testosterone treatment was correlated positively with the participants’ f0 and mean peak frequency of /s/. Crucially, testosterone should only affect f0, but not /s/, so changes in /s/ production would reflect the learned expression of masculinity. Zimman’s study shows that the acoustic features of f0 and /s/ do not necessarily vary systematically along a unidimensional continuum of “gender.” Rather, individuals modify a set of phonetic variables to express gender differently – to express different *gendered personae* – in different settings. Taken together, these studies show that gender represents a constellation of learned behaviours. The expression of gender is not simply sex-specific but rather socially learned and constructed by individuals. Zimman referred to this process as *stylistic bricolage*: constructing a novel gendered persona by using different combinations of speech features, some of which accord with cisnormative behaviour and some of which do not.

### 1.3. Gendered speech is learned in childhood

There is also emerging evidence that children express their gender identity through speech. Children’s vocal tracts do not show consistent sexual dimorphism before 8 years of age – i.e., there are no consistent differences in vocal-tract morphology by SAB until this age (Barbier et al., 2015; Vorperian et al., 2009). Therefore, gender differences found in children’s speech before this age cannot be solely attributed to sex differentiation in vocal-tract and/or laryngeal anatomy.

Nevertheless, numerous studies have shown that children under 8 years of age speak in ways that mirror the speech differences between adult cis men and women in their communities. While numerous perception studies have also shown that adults are able to perceive children’s SAB well above chance, even in children as young as 2.5 years of age (Barreda & Assmann, 2021; Fung et al., 2021; Munson et al., 2022; Perry et al., 2001), at least one recent study has found that some 6- to 7-year-old German-speaking children (46 out of 62 children that participated in the study by Funk & Simpson, 2023) were not unanimously perceived as *definitely a boy* or *definitely a girl* by the adult listeners. In the following section of the literature review and throughout this paper, we will use the terms AMAB and AFAB to refer to children’s gender, instead of the canonical terms *boys* and *girls.* This is because gender is a social construct and should not be imposed or assigned by others, consistent with the contemporary understanding of gender identities. Most past studies generally did not report whether children were asked for their gender identity, nor whether they or their caregivers were provided with options other than the binary gender of boy/male and girl/female.

Crucially, this line of research on children’s gendered speech found that AMAB and AFAB children learned to speak in gendered ways that are congruent with their SAB. T. L. Perry et al. (2001) examined single-word productions by children in four age groups (4, 8, 12, and 16 years of age) by asking adult listeners to rate the children’s speech along a six-point scale from *definitely a boy* to *definitely a girl.* Adult listeners were able to identify children’s SAB well above chance even for the youngest group. Fung et al. (2021) analysed longitudinal speech data from six AMAB and AFAB children at 2.5, 4, and 5.5 years old. They found that listeners correctly determined children’s SAB at greater than chance levels at even the youngest age. Munson et al. (2022) examined longitudinal data of 110 AMAB and AFAB children at 3 and 5 years old. They asked listeners to rate each child’s speech on a continuous scale anchored by the text *definitely a boy* and *definitely a girl.* The use of a continuous scale was intended in part to invite responses beyond the two SABs “male” and “female,” and hence is referred to as *perceived gender ratings* throughout this paper. Munson et al. found differences in perceived gender ratings for AMAB and AFAB children as young as 2.5 years. In interpreting these studies – including our own previous work on this topic – we work under the assumption that the statistical majority of people are cisgender: that the majority of AMAB children will likely adopt a male gender, and the majority of AFAB children will likely adopt a female gender. We concede that this does not accurately reflect the approximately 2% of children who are not cisgender (Kidd et al., 2021). The data analysed in this paper were not collected to examine gender development, and direct measures of gender identity were not available for these children.

Other researchers have also investigated gender differences in children’s sibilant sounds (Flipsen et al., 1999; Fox & Nissen, 2005; Li et al., 2016). These studies found that AMAB children produced /s/ with a lower spectral mean frequency than AFAB children, mirroring the pattern found in adult men and women. This is particularly compelling evidence of the learned nature of children’s gendered speech, given the wealth of findings that variation in adults’ /s/ cannot be traced entirely to vocal-tract variation.

Further evidence of children’s ability to express gender is shown in Cartei, Banerjee, et al. (2019), who examined the speech of children aged 6–10 years old in an acting task. Children were asked to impersonate characters varying in masculinity or femininity. They found that both boys and girls raised their f0 and formant frequencies when performing more feminine characters and lowered their f0 and formant frequencies when performing a more masculine character. Cartei et al.’s findings are powerful evidence that children have the capacity to express and construct gender.

Munson et al. (2015) provided evidence for the figurative performance of gender through speech. Munson et al. examined the speech of AMAB children with a diagnosis of Gender Identity Disorder (GID) and children without GID. GID is an obsolete diagnostic category, no longer present in the *Diagnostic and Statistical Manual of Mental Disorders* (DSM), the manual providing a uniform set of mental health conditions and diagnostic markers published by the American Psychiatric Association (2013). The criteria for GID included whether the child was displaying behaviours inconsistent with cisgender AMAB children (i.e., preferences for girl peers and for toys that are designed for and marketed toward girls). Listeners who were blind to children’s diagnostic status rated the boys with and without GID differently using the scale from Perry et al. (2001). Acoustic analysis of the children’s speech showed that the AMAB children with GID produced /s/ differently from their peers without GID, in ways that mirror the differences between adult women and men. These studies not only suggest that children manipulate the place of articulation of /s/ to express gender, but that this expression can vary within a single SAB as a function of nascent gender identity.

### 1.4. The ways that children mark gender phonetically are understudied

Previous research has provided compelling evidence that children produce gendered speech relatively early in life and that these features are perceptually salient to adult listeners. However, there is relatively little work on the specific *ways* that children mark gender in speech. A parallel line of research on the acoustics of gendered speech in German-speaking children was done by Simpson and colleagues. The authors collected a corpus of single-word, sentence-repetition, and continuous speech samples of children aged 6–10 years, as well as perceptual ratings of children’s perceived gender from 167 adult listeners. In Funk and Simpson (2023), the authors analysed the speech data of the 6-year-olds. In an acoustic analysis of a subset of children whose speech was rated as the most male- and female-sounding, substantial gender differences were found in f0, which also predicted the perceived gender ratings. Furthermore, their cluster analysis also showed that the speech of these two subsets of AMAB and AFAB children can be distinguished solely by the acoustic cue of f0, while another two subsets of children can be distinguished using a range of acoustic variables other than f0, such as vowel space size, sibilant acoustics, and speech rate. These results indicate that German-speaking children are also using a constellation of consonant and vowel acoustics to express and construct their gender.

Simpson et al. (2017) examined the speech of the 8- to 9-year-olds. AFAB children were found to speak with a faster speech rate than AMAB children, and their voices were characterized by a higher harmonics-to-noise ratio than the AMAB children. In another study by Funk et al. (2023), the authors found that 6- to 8-year-old AMAB children speak with a faster speech rate and a higher harmonics-to-noise ratio than the AFAB children, in contrast to their findings among the older group of children. The authors argued that the differences in speech rate are reflecting gender differences in reading competency, as the older group of children were reading written stimuli, whereas the younger group of children were repeating sentences that they heard in the production experiment. While there is a line of research focusing on German-speaking children, no study has examined variation in consonant and vowel production of 3- to 5-year-old English-speaking children in a single cohort, nor has any study examined English-speaking children’s gender marking longitudinally to understand how children’s gender expression changes over development.

Given the paucity of phonetic features that have been studied frequently, we have an incomplete picture of the phonetic cues that allow adult listeners to judge children’s SAB. The current study addresses these gaps by documenting and comparing phonetic markers of gender in a large set of single-word productions of the 110 children AMAB and AFAB from the longitudinal study by Munson et al. (2022), when the children were 3 years old and again when they were 5 years. It examines four measures that have been shown to differ between adult cisgender men and women, including f0, /s/ spectral centroid, vowel-space dispersion (VSD), and acoustic VTL (aVTL). We also examine whether these acoustic measures predict perceived gender ratings by adult listeners.

VSD is a measure of the size of the two-dimensional space of the first formant frequency by the second formant frequency. Larger vowel spaces are associated with more-distinct vowels and hence clearer speech (Bradlow et al., 1996). Women typically produce larger vowel spaces than men (Munson et al., 2006), and there is evidence that the size of the vowel space is manipulated volitionally to convey gender (Heffernan, 2010; Munson, 2007).

This study also examines a variable we call aVTL, the calculation of which is described in greater detail in the methods below. The length of the anatomical vocal tract is positively correlated with the average formant frequencies of vowels. The relationship between anatomical VTL and formant frequencies is described by simple principles of tube resonance (Chiba & Kajiyama, 1958). aVTL uses these principles to estimate VTL from vowels’ observed formant frequencies. Not surprisingly, Lammert and Narayanan (2015) showed that aVTL is correlated with measures of anatomical VTL taken from resting-state MRI (i.e., not while speaking). The imperfect nature of this correlation likely reflects different talkers’ use of articulatory manoeuvres to lengthen or shorten the vocal tract while speaking to convey socio-indexical information like gender. While this measure may reflect articulatory gestures across all vowels of a speaker, it should be noted that this measure is also contingent on the specific set of stimuli. This means that aVTL measurement is likely to vary across languages, dialects, and even studies with different sets of stimuli. A detailed explanation of this limitation of aVTL is illustrated in Barreda and Nearey (2018).

This study also examines correlations among the four measures. This analysis is important because if gender were monolithic, we would expect these measures to correlate strongly: being a woman means having a high f0, a dispersed vowel space, a high frequency /s/, and a short aVTL. But contemporary theories of gender and speech (Zimman, 2017) emphasize that gender is *not* monolithic: there are many ways of being male, female, or something else altogether – which is presumably why Zimman finds such weak correlations in the trans men he examines. If we find similarly weak correlations, it would provide further evidence of the non-monolithic nature of gender.

In sum, this paper examines four research questions:

1. How do children AMAB and AFAB differ for each of these measures (spectral centroid of /s/, aVTL, f0, and VSD)?
2. How do these measures change between 3 and 5 years of age?
3. How do these measures correlate with one another within individuals?
4. How do these measures predict adult listeners’ ratings of the perceived gender of these children’s speech reported in Munson et al. (2022)?

The descriptive data from questions 1 and 2 will help us better understand the different ways that children learn to mark gender during the preschool years. The data from question 3 help us to understand whether children’s gender marking is monolithic (i.e., all features associated with cisgender adult men correlate with one another within an individual speech style) or whether there are dissociations among measures. The data from question 4 will allow us to understand the features that are most important in adults’ appraisals of children’s gender through speech. The analyses for question 4 resemble those in Munson et al. (2022), which examined how /s/ acoustics and f0 of the small set of children’s productions used in a rating task predicted listeners’ ratings. Only four productions from each child – those that were used as stimuli in that paper’s perception experiment – were analysed. Moreover, only two of these tokens contained word-initial /s/. Not surprisingly, the phonetic diversity of these tokens was quite restricted. The current paper analyses the full set of speech tokens, an average of 68 vowels and 11 /s/ tokens for each child at each time point (TP). These were collected from children as part of their participation in the larger longitudinal study on relationships between phonological development and vocabulary growth. The result is a much more robust characterization of the phonetic characteristics of these children’s speech, including measures like VSD or aVTL that could not be made confidently with the small number of tokens analysed in Munson et al. These measures are used to predict the ratings from Munson et al. (2022) to determine whether the predictors of the ratings in that study hold when a more thorough assessment of these children’s speech is provided. We reasoned that the mean gender rating for each child is an index of children’s habitual gender expression through speech. Given this, using a larger set of acoustic measures to predict gender ratings reflects the acoustic characteristics that affect adults’ appraisal of children’s gender in daily contexts, rather than the influence of token-by-token variation on isolated appraisals of gender.

## 2. Methods

### 2.1. Participants

The speech corpus examined in this study contains the audio recordings of the 110 children reported in Munson et al. (2022). The children participated in a longitudinal study on phonological development and vocabulary growth and did not focus specifically on gender or sociolinguistic learning. Consistent with the goals of that study, the gender identity of the children was not asked at any recording sessions, so we grouped children according to SAB rather than gender, a point we return to in the discussion. Specifically, the child talkers are 55 AMAB and 55 AFAB children, individually matched for age +/−3 months at the time of recording. All child talkers participated in a speech-production experiment at two TPs: 28 to 39 months of age (which we call the first TP, or FTP, as in Munson et al., 2022) and 53 to 66 months of age (last TP, LTP). The broad age range within TPs was an intentional choice given the goals of the original study. An additional TP intermediate between these two is not analysed in this paper. All children passed a binaural hearing screening at 25 dB at octave frequencies between 0.5 and 8 kHz. One hundred and four of the children (94.5%) were exposed to a local white dialect of American English (spoken in the Twin Cities, MN metropolitan area, or Madison, WI) growing up, and 6 were exposed to African American English.

### 2.2. Data collection

At each recording session, the child talkers participated in a word repetition task, in which they were presented with an image of a familiar word on a screen in front of them and simultaneously heard a pre-recorded auditory prompt (e.g., “Chair!”) presented in free field. The children repeated the word. Children were provided with additional repetitions and cues if needed. Words were presented over loudspeakers at a comfortable level. The order was pseudo-randomized, such that there were no repetitions of words on consecutive trials. Children’s productions were recorded with a Shure SM81 cardioid condenser microphone on a Marantz PMD 671 solid-state recorder, with a 44.1 kHz sampling rate. The best production for each trial was chosen by a trained research assistant, using a procedure described in detail in Munson et al. (2022).

There were approximately 100 test words at each recording session. The exact number differed by TP since there were more candidate words for the older children, and they had longer attention spans. The words were chosen to sample selected consonant contrasts word-initially in balanced vowel contexts that were balanced for height and backness. The words were picturable, and with an age of acquisition based on Kuperman et al. (2012). Some words were repeated to elicit sufficient tokens of vowels and consonants. The full list of the test words used is displayed in Table 1. These reference transcriptions reflect pronunciation patterns in the region where the study took place, where, for example, people do not produce a consistent distinction between COT and CAUGHT (Labov et al., 2008). The experiment stimuli were recorded words produced by two trained female research assistants, one of whom produced the stimuli in the local white American English variety, and the other in African American English. Children received the stimuli from their home dialect.

**Table 1.** Full list of test words at the first time point and last time point, separated by vowels. Asterisks denote words that were used as stimuli in the perceived gender rating study. Words that were used for /s/ analysis are italicized Table 1. long description.

First time point				
/i/ (n = 293)	/ɪ/ (n = 1366)	/ɛ/ (n = 831)	/æ/ (n = 1019)	/u/ (n = 666)
sheep /ʃip/	dinner /ˈdɪnɚ/ dish /dɪʃ/ kitchen /ˈkɪʧən/ kitty /ˈkɪɾi/ scissors* /ˈsɪzɚz/ tickle /ˈtɪkəl/ give /ɡɪv/ *sick* /sɪk/	share /ʃɛr˞/ teddy bear /ˈtɛdibɛr/ get /ɡɛt/	candy/ˈkændi/ cat /kæt/ daddy /ˈdædi/ dance /dæns/ *sandwich* /ˈsændwɪʧ/ *sad* /sæd/	tooth /tuθ/ shoe /ʃu/ *soup* /sup/
/ʊ/ (n = 318)	/ʌ/ (n = 1204)	/ɑ/ (n = 1039)	/oʊ/ (n = 133)	
cookie* /ˈkʊki/ good /ɡʊd/	*sun* /sʌn/ cup /kʌp/ shovel* /ˈʃʌvəl/ tongue /tʌŋ/ tummy /ˈtʌmi/ duck /dʌk/ gum /ɡʌm/	car /kɑr/ dog /dɑɡ/ or /dɔɡ/ door /dɑr/ or /dɔr/ garbage* /ˈɡɑrbɪʤ/ *sock* /sɑk/	*soap* /soʊp/	
Last time point				
/i/ (n = 687)	/ɪ/ (n = 1053)	/ɛ/ (n = 1218)	/æ/ (n = 1167)	/u/ (n = 737)
cheese /ʧiz/ keys /kiz/ reading /ˈridɪŋ/ teacher /ˈtiʧər/ queen /kwin/ sheep /ʃip/ wheel /wil/	*cereal* /ˈsɪriəl/ chicken /ˈʧɪkən/ kitchen /ˈkɪʧən/ kitten /ˈkɪtn/ *scissors* /ˈsɪzərz/ *sink* /sɪŋk/ *sister* /ˈsɪstər/ tickle /ˈtɪkəl/ window /ˈwɪndoʊ/ ship /ʃɪp/	chair /ʧɛr/ red /rɛd/ *seven* /ˈsɛvən/ sharing /ˈʃɛrɪŋ/ teddy bear /ˈtɛdibɛr/ twelve /twɛlv/ twenty /ˈtwɛnti/ web /wɛb/ shell /ʃɛl/ tent /tɛnt/ wet /wɛt/	candle /ˈkændl/ candy /ˈkændi/ cracker /ˈkrækər/ rabbit* /ˈræbɪt/ *sandbox* /ˈsændbɑks/ *sandwich* /ˈsændwɪʧ/ shadow /ˈʃædoʊ/ cat /kæt/ trash /træʃ/	roof /ruf/ room /rum/ shoes /ʃuz/ *suitcase* /ˈsutkeɪs/ toothbrush /ˈtuθbrʌʃ/ *soup* /sup/
/ʊ/ (n = 605)	/ʌ/ (n = 1539)	/ɑ/ (n = 710)	/aɪ/(n = 86)	
cookie* /ˈkʊki/ sugar /ˈʃʊɡər/ wolf /wʊlf/ woman /ˈwʊmən/ wood /wʊd/	cousin /ˈkʌzən/ cutting /ˈkʌɾɪŋ/ running /rʌnɪŋ/ summer* /ˈsʌmər/ tongue /tʌŋ/ trouble /ˈtrʌbəl/ tummy /ˈtʌmi/ *sun* /sʌn/ cup /kʌp/ shovel* /ˈʃʌvəl/ truck /trʌk/	coffee /ˈkɑfi/ crawl /krɑl/ or /krɔl/ walk /wɑk/ washcloth /ˈwɑʃklɔθ/ washer /ˈwɑʃər/ water /ˈwɑɾɚ/ or /ˈwɔɾɚ/ rock /rɑk/	*sidewalk* /ˈsaɪdwɑk/	

### 2.3. Acoustic analysis

Audio recordings of the children’s word productions were segmented using Praat (Boersma & Weenink, 2022). If there were two responses from the child talker for one prompt, the best production was selected for acoustic analysis, based on an annotator’s judgment on accuracy and clarity of pronunciation. The productions that were not the lexical target were removed from further analysis.

***Analysis of /s/.*** A subset of the original test words (N = 16 for each child) was selected for the acoustic analysis of /s/ (the italicized words in Table 1). The /s/ sounds were always word-initial, in a stressed syllable, and immediately prior to a vowel.

The word-initial /s/ sounds were segmented manually for acoustic analysis. The onset of /s/ was defined as the onset of high-frequency turbulent noise in the spectrogram and aperiodicity in the waveform. The offset of /s/ was defined as the beginning of voicing in the subsequent vowel. Tokens that were judged as erroneous pronunciation, either for incorrect manner of articulation or incorrect place of articulation, together with those that were unable to extract spectral centroid due to technical complications (i.e., produced in the presence of background noise or overlapping speech), were excluded from subsequent analysis. Ten children did not have any usable /s/ tokens at FTP, and one child did not have any usable /s/ tokens for both FTP and LTP.

We employed a Praat script to measure the spectral centroid of each /s/ token (the mean frequency, in Hz). The spectral centroid of /s/ was calculated for the middle 40 ms interval of frication. We band-pass filtered this with a lower cutoff of 500 Hz to remove any artifacts of coarticulatory voicing. As in Li and Munson (2016), we used multitaper spectra. Tokens with spectral centroids that exceeded +/−2 SD from the mean across all children were removed from subsequent analysis. As a result, there were 980 tokens of /s/ in FTP and 1310 tokens in LTP. ***Analysis of Vowels.*** We analysed the monophthongal vowels /i ɪ ɛ æ u ʊ ɔ ʌ ɑ/ in the stressed position of the test words (see Table 1 for the word list). Initial segmentation and alignment were conducted using the Montreal Forced Aligner (McAuliffe et al., 2017) and were manually verified by the first author. Words that contained a consonant misarticulation were included in the analysis of vowel acoustics.

Due to the high f0 of children’s speech, formant tracking is difficult. The widely spaced harmonics in the sound source spectrum may misalign with the peaks of the formants in the vocal-tract resonance function. A customized Python script, *Triple Formant Tracker*, adopted from Cychosz (2020), was used to measure formant frequencies of the vowels to mitigate this potential difficulty. Formant frequencies were measured at the temporal midpoint using three algorithms: Inverse Filter Control Formant (Watanabe, 2001), Entropic Signal Processing Systems’ (ESPS) covariance, and ESPS’s autocorrelation. A median formant measurement for each token was then computed from these values. For the two ESPS algorithms, the order of Linear Predictive Coding was set as 10, allowing three formants to be extracted; the nominal F1 was 700 Hz. F0 was measured automatically through the IFC algorithm in the *Triple Formant Tracker* by sampling at the midpoint of each vowel. We examined the raw data and removed any tokens with either f0, F1, F2, or F3 that exceeded +/−2 SD from the mean for each vowel phoneme across all children. This procedure mostly eliminated the vowel tokens with extremely high f0 values. Subsequently, a total of 7201 tokens in FTP and 7,856 tokens in LTP remained in the analysis.

To compute aVTL, we first obtained the mean F1, F2, and F3 of each vowel category and the ratio between each adjacent pair of formants (ΔF) for each child at each TP, using the average formant spacing formula in Johnson (2020), where ΔF = (MeanF1 * 0.5 + MeanF2 * 1.5 + MeanF3 * 2.5) /3. This measure results in a single scalar representing the constant distance between a speaker’s formants (F1 to F2, F2 to F3, etc.). We used the mean formant frequencies of each vowel category to derive ΔF to avoid this measurement being skewed by the missing or unbalanced number of tokens across different vowels, an issue illustrated in Barreda and Nearey (2018). The aVTL (in cm) was then estimated using the formula: aVTL = 34000/2*ΔF. As demonstrated in Johnson (2020), this measure of aVTL correlates with the VTL data measured from MRI in Lammert and Narayanan (2015).

To measure VSD, we first normalized the three formant frequencies using the ΔF value to eliminate inter-child VTL differences for a fair comparison of their VSD (e.g., normalized F1 = F1 (in Hz)/ ΔF). As such, measurements of VSD without normalization might simply indicate individual differences in vocal-tract sizes between FTP and LTP. Upon normalization of vowel formant frequencies, we measured the centre of the F1 by F2 vowel space for each child by taking an average of the normalized F1 values and the normalized F2 values. We then measured the Euclidean distances between each vowel token and the centre of the F1 by F2 vowel space for each child. The equation is displayed as follows:

$$\sqrt{{\left(mean\backslash ;F1-token\backslash ;F1\right)}^{2}+{\left(mean\backslash ;F2-token\backslash ;F2\right)}^{2}}$$

**Figure 1.**
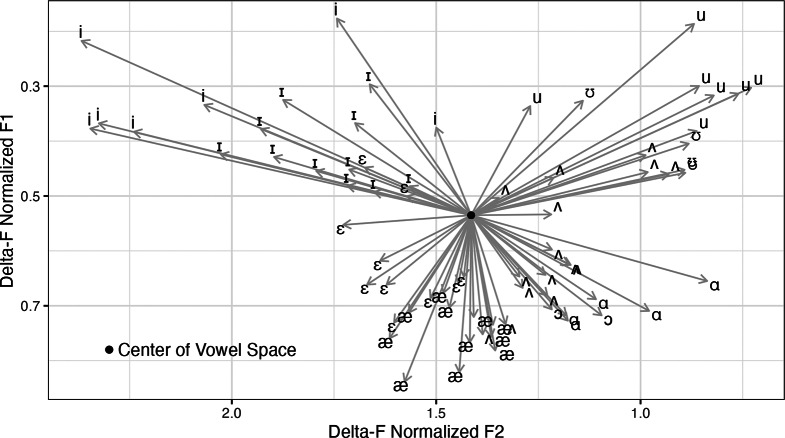
Illustration of vowel-space dispersion (VSD) measurement in one child. VSD is taken as the mean of the Euclidean distances between the centre of the ΔF normalized F1 by F2 vowel space and all vowel tokens. Figure 1. long description.

The measurement of Euclidean distances for one child in the dataset is illustrated in Figure 1. VSD is taken as the average of the Euclidean distances of each vowel token from the centre of the vowel space.

### 2.4. Adult listeners’ ratings of children’s perceived gender

The adult listeners’ ratings of children’s perceived gender were obtained from Munson et al. (2022). A full description of the procedures for collecting these ratings is given in that study. The ratings were made by 80 individual adults who were native speakers of English with no speech, language, or hearing impairments. Children’s speech samples were divided into four sets of approximately equal size; the stimuli in each of these sets were rated by groups of 20 adults. This decision was made to ensure that the experiment length was manageable. In the experiment, participants heard eight productions from each child: four from FTP (cookie, shovel, garbage, and scissors) and four from the LTP (cookie, shovel, rabbit, and summer). The stimuli used for eliciting perceived gender ratings were marked with an asterisk in Table 1. Stimulus presentation was blocked by age and randomized within age.

The adult listeners were asked to rate the child’s gender on a continuous visual analog scale comprising a double-headed arrow anchored by the text *definitely a boy* at one end and *definitely a girl* at the other. The click location along the arrow (in pixels) was recorded. A total of 18,580 ratings were elicited (FTP = 9280 ratings; LTP = 9300 ratings).

### 2.5. Statistical analysis

Statistical analyses were conducted using the *lme4* and *glmmTMB* packages (Brooks et al., 2017) in R version 4.4.1 (RStudio Team, 2020). These analyses were complicated by the fact that two of our measures, aVTL and VSD, are by definition aggregated across items. The other two, f0 and spectral centroid of /s/, do not require aggregation. Hence, for some of our analyses, we calculated averages for /s/ spectral centroid and f0 so that they could be compared directly to aVTL and VSD.

We first evaluated how the four acoustic variables (aVTL, VSD, f0, and /s/ spectral centroid) were correlated with each other. We measured the Pearson’s r correlations among aVTL, VSD, mean f0, and mean /s/ spectral centroid. The p-values were Bonferroni-corrected. We then examined the phonetic differences between AMAB and AFAB children, where we fitted linear mixed-effect models to predict aVTL, VSD, f0, and /s/ spectral centroid. Each model began with the fixed effects of TP and SAB. For the model predicting VSD, an additional fixed effect of mean vowel duration was added to control for the effect of speaking rate on VSD (Moon & Lindblom, 1994). We then tested if adding the interaction between TP and SAB would improve model fit using likelihood tests. As for random effects, the models predicting aVTL and VSD included a random intercept by child. For the models predicting f0 and /s/ spectral centroid, the random slopes of TP by child as well as TP and SAB by word items were included. The random effects were pruned based on the smallest variance reported in model output in R until the model converged. For all statistical analyses, the TP of FTP and LTP was contrast coded as −1 and 1, respectively. For SAB, AMAB and AFAB were coded as −1 and 1, respectively.

To examine whether the four acoustic variables (i.e., aVTL, VSD, f0, and /s/ spectral centroid) predict perceived gender ratings, we fitted two generalized mixed models with a beta response distribution and a logit link function. We used the beta_family function in glmmTMB as the perceived gender ratings are bounded variables. We first transformed the perceived gender ratings from the original scale of 460 to 1460 pixels to a numerical scale from 0.00004 to 0.99996 for statistical analysis. This was because the beta_family function did not allow the response variable containing data values of absolute 0 nor 1. In these models, the dependent measures were the perceived gender ratings. All available ratings were used in the statistical analysis. The predictors were aVTL, VSD, mean f0, and mean /s/ spectral centroid, which were all rescaled from 0 to 1 to allow a direct comparison of their effects on the ratings.

The first model evaluated if aVTL, VSD, and mean f0 predict gender ratings. These acoustic features were included in one model to account for the possible correlation between these vowel features. Moreover, studies of gender perception often show that these acoustic features are used jointly to determine a speaker’s gender (e.g., (Barreda & Assmann, 2021)). Therefore, having one single model would allow us to directly compare the effects of these vowel acoustic features on perceived gender ratings. The initial model included an interaction of TP and SAB and the random intercepts of rater, child, and word. We then added the fixed predictors of aVTL, VSD, and mean f0, and their interactions with TP and SAB through a forward testing procedure. Predictors that did not improve model fit based on likelihood tests were removed from the model. Then, the maximal random effect structure was also added. The random effects were pruned in a stepwise manner, based on the variance in the model output, until the model converged.

The second model evaluated whether /s/ spectral centroid predicts gender ratings. The response variables were only the subset of ratings elicited by the /s/−initial stimuli, i.e., “scissors” (used in FTP) and “summer” (used in LTP). The initial model included the interaction effect of TP and SAB. The random effects were the intercepts of rater, child, and word. We then added the fixed effect of mean /s/ spectral centroid and its interaction with TP and SAB in a forward testing procedure. Model fit was determined by likelihood tests. We then included the maximal random effect structure, and the random effects were pruned in a stepwise manner until the model converged. It should be noted that Munson et al. (2022) did analyse the relationships between perceived gender ratings and /s/ spectral centroid and f0. Again, we acknowledge that the analysis of /s/ acoustic and perceived gender ratings is not entirely independent from Munson et al. (2022). Here, we re-analysed a larger set of data from Munson et al. (2022) to examine whether the relationship between /s/ acoustic and perceived gender rating could be replicated with a more robust characterization of children’s /s/ production.

## 3. Results

### 3.1. Correlations between the acoustic variables

We first examine whether the four acoustic variables were correlated with each other. The Pearson’s *r* correlations between the four scaled acoustic variables are shown in Table 2 for FTP and Table 3 for LTP. The correlation of aVTL and mean /s/ spectral centroid was significant at both TPs. The negative correlation coefficients at FTP and at LTP suggest that children with “longer” aVTL also had lower mean /s/ spectral centroid (FTP: *r*(97) = −.29, *p* = .02; LTP: *r*(106) = −.39, *p* < .001). This direction is consistent with differences between adult men and women in previous studies. A longer aVTL would likely be perceived as male-sounding, as cisgender men typically have longer vocal tracts than women. On the other hand, the lower /s/ spectral centroid was indicative of a less anterior constriction (i.e., a less fronted /s/), which is typically associated with cisgender men in the literature. One possibility is that these measures reflect co-occurring learned behaviours that convey gender. Another is that it reflects the influence of actual VTL on both of these measures. That latter interpretation is inconsistent with the findings of Fuchs and Toda (2010). Moreover, the strength of the correlation between aVTL and /s/ spectral centroid does not necessarily increase over time, as the confidence intervals of r values overlap between the two TPs (FTP: −.46 to −.10; LTP: −.54 to −.22). The correlation of VSD and mean f0 at LTP was also significant, *r*(108) = .25, *p* = .04. This suggests that a higher f0 is associated with a larger VSD when the children were 5 years of age. These features are both congruent with the finding of gender differences in American English-speaking cisgender adults. For example, Munson and Solomon (2016) found that cisgender women tend to speak with more dispersed vowel space than cisgender men. To avoid overlooking strong curvilinear relationships between the acoustic variables, we have also visualized each pair of acoustic variables. However, we did not observe any strong curvilinear relationship. These scatterplots can be accessed through the Supplementary Appendix. Overall, the relatively weak correlations between the four acoustic variables at both TPs suggest that the development of these gendered phonetic features was not parallel in children of the current study.

**Table 2.** Correlation matrix with confidence intervals of the four scaled acoustic variables at the first time point Table 2. long description.

	aVTL^a^	VSD^b^	Mean f0^c^	Mean /s/^d^
VSD	0.24	—		
	[0.05, 0.41]			
Mean f0	0.04	0.12	—	
	[−0.15, 0.23]	[−0.07, 0.30]		
Mean /s/	−0.29^*^	0.18	0.15	—
	[−0.46, −0.10]	[−0.02, 0.36]	[−0.05, 0.34]	

a Acoustical Vocal-Tract Length.

b Vowel-space dispersion.

c Mean fundamental frequency averaged across tokens.

d Mean of /s/ spectral centroid averaged across tokens.

* indicates *p* < .05.

**Table 3.** Correlation matrix with confidence intervals of the four scaled acoustic variables at the last time point Table 3. long description.

	aVTL^a^	VSD^b^	Mean f0^c^	Mean /s/^d^
VSD	0.16	—		
	[−0.03, 0.33]			
Mean f0	−0.06	0.25^*^	—	
	[−0.24, 0.13]	[0.07, 0.42]		
Mean /s/	−0.39^***^	0.003	0.23	—
	[−0.54, −0.22]	[−0.19, 0.19]	[0.05, 0.40]	

a Acoustic Vocal-Tract Length.

b Vowel-space dispersion.

c Mean of fundamental frequency averaged across tokens.

d Mean of /s/ spectral centroid averaged across tokens.

* indicates *p* < .05.

*** indicates *p* < .001.

### 3.2. Phonetic differences between AFAB and AMAB children

**Figure 2.**
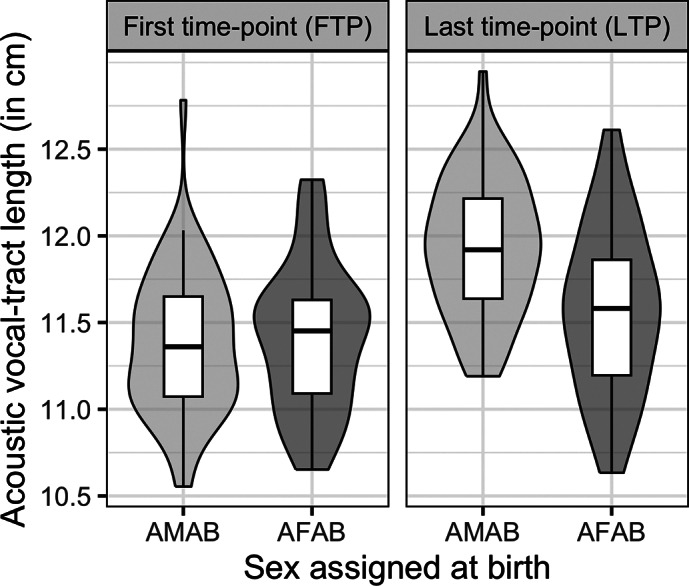
Violin plots of acoustic vocal-tract length of the 110 children, separated by sex assigned at birth and time points. Figure 2. long description.

To examine whether the four acoustic variables (aVTL, VSD, f0, and /s/ spectral centroid) differ between TPs and SAB, we fitted four separate linear mixed-effect models. For aVTL, the best-fitted model showed a significant interaction effect of TP and SAB (β = −.10, *z* = −4.46, *p* < .001). Table 4 shows the full model statistics. Figure 2 displays the violin plots and box plots of the children’s aVTL separated by SAB and TPs. As shown in Table 4, the negative slope of SAB suggests that AMAB children had longer aVTL than AFAB children. A post-hoc analysis showed (i) a significant gender difference in aVTL at LTP (*p* < .001), but not at FTP (*p* = .69); (ii) the gender difference in aVTL is driven by the larger increase of aVTL from 3 to 5 years of age among the AMAB children (*p* < .001), compared to AFAB children (*p* = .05). Overall, our results suggest that gender differences in aVTL were present at 5 years of age.

**Table 4.** Linear mixed-effect model predicting acoustic vocal-tract length from time point and sex assigned at birth, with a random intercept of child Table 4. long description.

	Estimate	SE	z	*p*	95% CI
Intercept	11.57	0.03	342.41	<0.001	11.50 to 11.64
Time point (FTP = −1, LTP = 1)	0.18	0.02	7.79	<0.001	0.13 to 0.22
SAB (AMAB = −1, AFAB = 1)	−0.09	0.03	−2.54	0.013	−0.15 to −0.02
Time point*SAB	−0.10	0.02	−4.46	<0.001	−0.15 to −0.06

**Figure 3.**
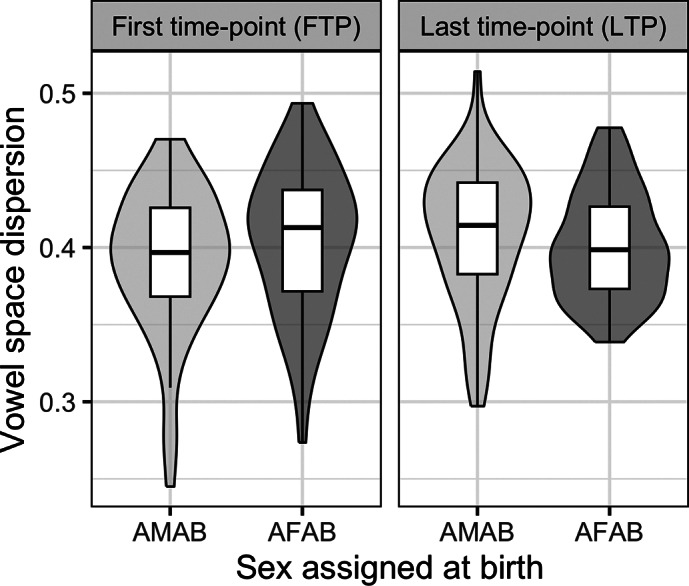
Violin plots of vowel-space dispersion of the 110 children, separated by sex assigned at birth and time points. Figure 3. long description.

The model predicting VSD showed no significant effect of TP, SAB, nor mean vowel duration (Table 5). The null effect suggests that we could not find evidence of gender difference in vowel space sizes in our dataset. However, it is important not to over-interpret this null result: our effect size is relatively robust, and the current study is relatively high-powered, but the null results cannot conclude the absolute lack of an effect. The distribution of VSD data is illustrated in Figure 3.

**Table 5.** Linear mixed-effect model predicting vowel-space dispersion from time point, sex assigned at birth, and mean vowel duration, with a random intercept of child Table 5. long description.

	Estimate	SE	z	*p*	95% CI
Intercept	0.40	0.02	21.16	<0.001	0.36 to 0.43
Time point (FTP = −1, LTP = 1)	0	0	1.26	0.21	0 to 0.01
SAB (AMAB = −1, AFAB = 1)	0	0	0.43	0.67	0 to 0.01
Mean vowel duration	0.02	0.09	0.24	0.81	−0.16 to 0.20

**Figure 4.**
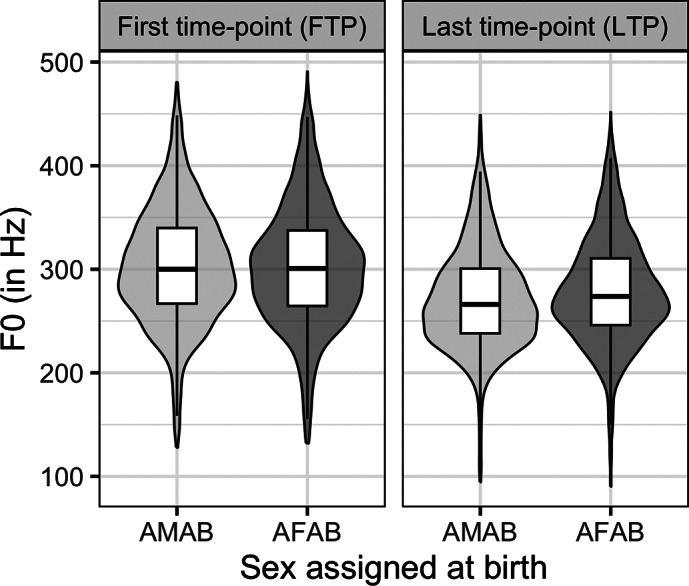
Violin plots of the fundamental frequency of the 110 children, separated by sex assigned at birth and time points. Figure 4. long description.

As for f0, the model showed a significant effect of TP (β = −14.86, *z* = −6.91, *p* < .001). This negative slope suggests that f0 decreased from the FTP to the LTP, which is consistent with previous studies of children in this age range (Glaze et al., 1988). There was no evidence of gender differences in f0. The full model statistics are shown in Table 6. The distribution of the f0 data is illustrated in Figure 4. Although f0 is arguably one of the most salient speech features that is sex dimorphic in cisgender adult men and women (Childers & Wu, 1991), the null effect in gender differences in prepubertal children’s f0 in the current study is consistent with findings in English-speaking children from previous studies (Fung et al., 2021; Perry et al., 2001).

**Table 6.** Linear mixed-effect model predicting fundamental frequency (f0) from time point and sex assigned at birth, with random slopes of time point by child and time point by word item Table 6. long description.

	Estimate	SE	z	*p*	95% CI
Intercept	289.91	3.09	93.81	<0.001	283.82 to 296.01
Time point (FTP = −1, LTP = 1)	−14.86	2.15	−6.91	<0.001	−19.12 to −10.61
SAB (AMAB = −1, AFAB = 1)	1.54	2.41	0.64	0.53	−3.24 to 6.31

**Figure 5.**
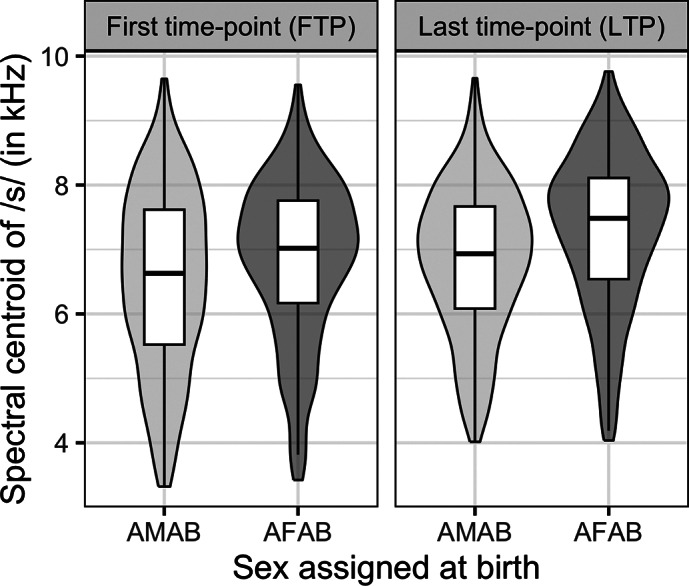
Violin plots of /s/ spectral centroid of the 110 children, separated by sex assigned at birth (SAB) and time points. Figure 5. long description.

As for /s/ spectral centroid, there was a significant effect of TP (β = 232.84, *z* = 4.39, *p* < .001) and SAB (β = 210.18, *z* = 2.92, *p* = .004). Full statistics of this model are shown in Table 7. The violin plot of children’s /s/ spectral centroid is shown in Figure 5. The positive slope of SAB indicates that AMAB children had lower /s/ spectral centroids than the AFAB counterparts for both TPs, which mirrors the gender difference in adults reported in the literature (Munson, 2007; Stuart-Smith, 2007). This suggests that children as young as 2.5 years of age were producing a variation of /s/ that matched the patterns found for the presumably cisgender adults in Jongman et al. (2000).

**Table 7.** Generalized mixed-effect model predicting /s/ spectral centroid from time point and sex assigned at birth, with random slopes of time point by child and sex assigned at birth by word item Table 7. long description.

	Estimate	SE	z	*p*	95% CI
Intercept	6731.06	79.26	84.92	<0.001	6573.75–6888.37
Time point (FTP = −1, LTP = 1)	232.84	53.10	4.39	<0.001	127.66–338.03
SAB (AMAB = −1, AFAB = 1)	210.18	71.93	2.92	0.004	67.34–353.01

To summarize the results thus far, we found gender differences in the aVTL and /s/ spectral centroid in AMAB and AFAB children. Differences between AMAB and AFAB children in aVTL were present at 5 years of age, whereas differences in /s/ were present at 3 years of age. We found no evidence of f0 and VSD differences between AMAB and AFAB children prior to 5 years of age.

### 3.3. Predicting perceived gender ratings

**Figure 6.**
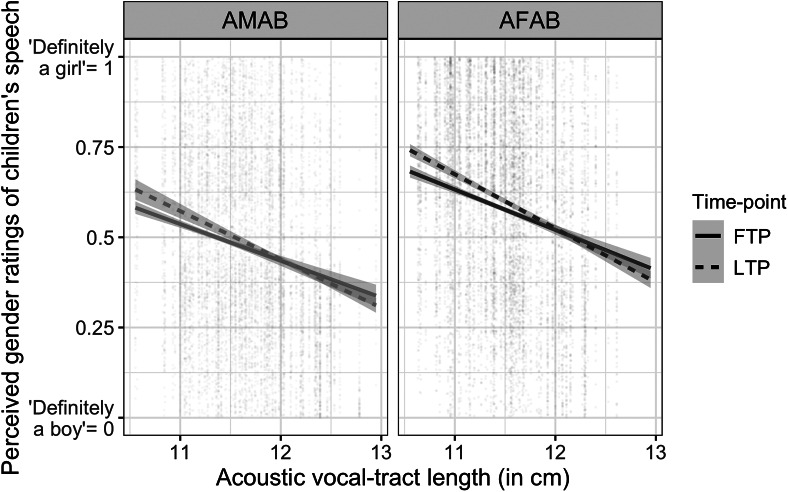
Line plot showing children’s perceived gender ratings predicted by sex assigned at birth, time points, and acoustic vocal-tract length. Figure 6. long description.

We next examined which of the four acoustic variables predict perceived gender ratings of children’s speech. We first fitted a generalized mixed model with a beta distribution to predict individual perceived gender ratings from aVTL, VSD, and mean f0. The best-fitted model (Table 8) showed a significant main effect of aVTL on perceived gender ratings (aVTL: β = −1.15, *z* = −5.51, *p* < .001). A longer aVTL was associated with lower perceived gender ratings (i.e., more “boy-like”), which was parallel to the sex-dimorphic aVTL differences in cisgender men and women. There was no significant interaction of aVTL, TP, and SAB effect on perceived gender ratings. The relationship between gender ratings, aVTL, TP, and SAB is illustrated in Figure 6. As illustrated in this figure, aVTL appears to be a robust cue in predicting perceived gender ratings of both AMAB and AFAB children across TP and SAB.

**Table 8.** Generalized mixed-effect model of perceived gender ratings predicted by sex assigned at birth, time points, and acoustic vocal-tract length, vowel-space dispersion, and mean fundamental frequency. Formula = Rating ~ time point* SAB* Mean f0 + aVTL + VSD + (0 + SAB: Mean f0 + aVTL + VSD | rater) + (SAB: Mean f0 + aVTL + VSD | child) + (aVTL + VSD | word) Table 8. long description.

	Estimate	SE	z	*p*	95% CI
Intercept	0.33	0.16	2.04	0.041	0.01 to 0.64
Time point (FTP = −1, LTP = 1)	−0.06	0.05	−1.12	0.262	−0.16 to 0.04
SAB (AMAB = −1, AFAB = 1)	−0.08	0.07	−1.13	0.258	−0.22 to 0.06
Mean f0	0.15	0.13	1.12	0.264	−0.11 to 0.4
aVTL	−1.15	0.21	−5.51	<0.001	−1.56 to −0.74
VSD	0.45	0.23	1.98	0.047	0.01 to 0.89
Time point*SAB	0.19	0.05	3.52	<0.001	0.08 to 0.29
SAB* Mean f0	0.41	0.13	3.13	0.002	0.16 to 0.67
Time point* Mean f0	0.06	0.10	0.61	0.545	−0.13 to 0.25
Time point*SAB* Mean f0	−0.24	0.10	−2.56	0.011	−0.43 to −0.06

**Figure 7.**
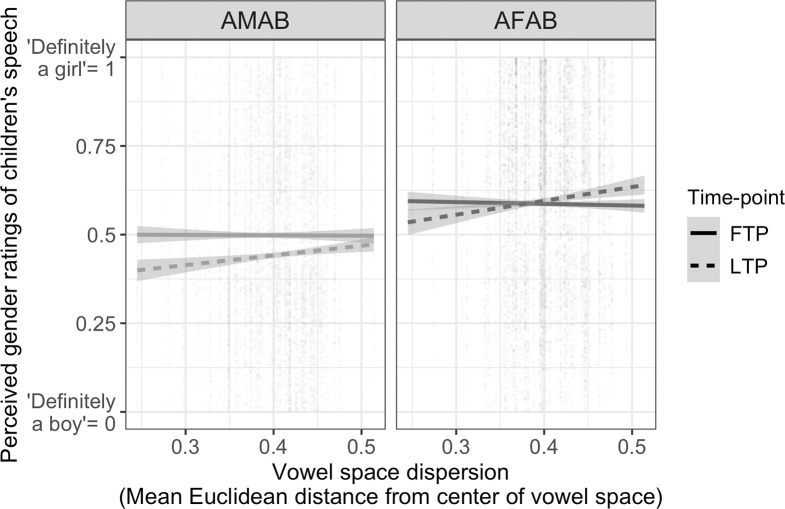
Line plot showing the relationship between perceived gender ratings of children’s speech by sex assigned at birth, time point, and vowel-space dispersion. Figure 7. long description.

There was also a significant main effect of VSD on perceived gender ratings (β = .45, *z* = 1.98, *p* = .047). The relationship between perceived gender ratings and VSD is illustrated in the line plot in Figure 7. The positive slope of VSD suggests that a more expanded or dispersed vowel space was associated with ratings toward more “girl-like.” This is consistent with the literature showing that cisgender women were perceived to speak with a more expanded vowel space than cisgender men (Munson et al., 2006). Although it appears that there was a stronger relationship between VSD and perceived gender ratings in LTP than FTP, as shown in Figure 7, there was no significant interaction effect of VSD and TP on the ratings.

**Figure 8.**
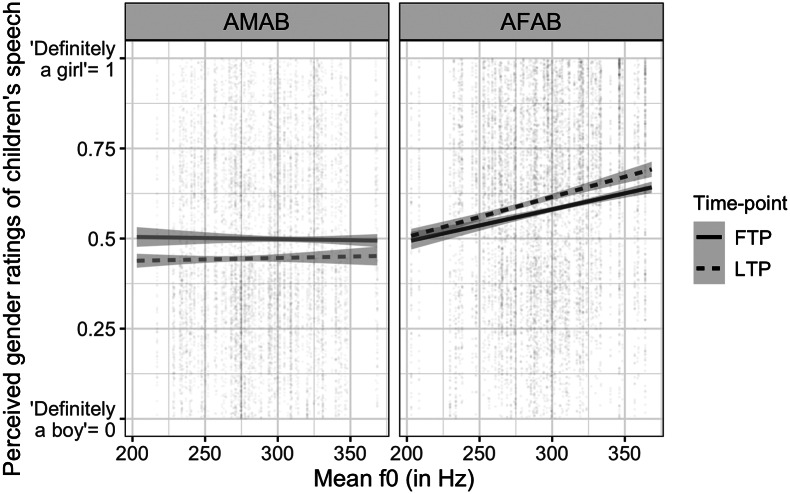
Line plot showing perceived gender ratings of children’s speech predicted by sex assigned at birth, time points, and mean fundamental frequency. Figure 8. long description.

As for f0, the model showed a significant interaction effect of TP, SAB, and mean f0 on predicting perceived gender ratings (β = −.24, *z* = −2.56, *p* = .01). The relationship between mean f0, TP, and SAB on perceived gender rating is illustrated in Figure 8. The negative slope suggests that the effect of mean f0 was stronger at LTP than FTP. The effect was also stronger for predicting the perceived gender ratings of AFAB children than those of AMAB children. The nature of this interaction effect is illustrated in Figure 8.

Overall, our analysis showed that the three acoustic variables (aVTL, VSD, and mean f0) all played a role in the perception of children’s gender. Among these variables, the fixed effect of aVTL had a larger absolute coefficient than that of VSD and mean f0, indicating that aVTL plays a major role in adults’ appraisal of children’s gender speech.

**Figure 9.**
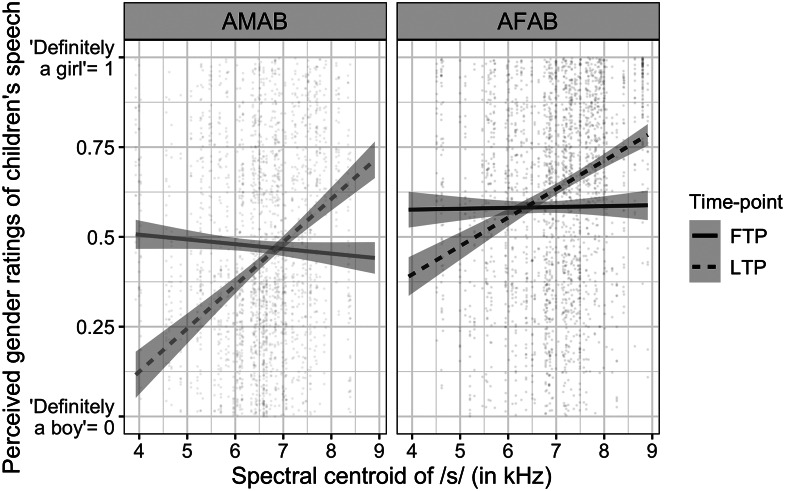
Line plot showing children’s perceived gender ratings of /s/−initial words predicted by sex assigned at birth, time points, and mean spectral centroid of /s/. Figure 9. long description.

Next, we fitted a generalized linear mixed-effect model to predict the perceived gender ratings of /s/−initial words. The best-fitted model (Table 9) showed a significant interaction effect of TP*SAB*mean /s/ spectral centroid on predicting perceived gender ratings (β = −.43, *z* = −3.02, *p* = .003). Figure 9 displays the relationship between mean /s/ spectral centroid, TP, SAB, and perceived gender ratings. A higher /s/ spectral centroid was associated with a more “girl-like” gender rating. The negative slope of the interaction effect suggests that the effect of /s/ acoustics on the perceived gender ratings was stronger in LTP than in FTP and a stronger effect for AMAB children than the AFAB children.

**Table 9.** Generalized mixed-effect model of perceived gender ratings of /s/−initial words predicted by sex assigned at birth, time points, and mean /s/ spectral centroid. Formula = /s/ Rating ~ Time point * SAB * Mean /s/ spectral centroid + (1 | rater) + (Mean /s/ spectral centroid | child) + (0 + Mean /s/ spectral centroid | word) Table 9. long description.

	Estimate	SE	z	*p*	95% CI
Intercept	−0.96	0.25	−3.82	<0.001	−1.45 to −0.47
Time point	−0.57	0.08	−6.88	<0.001	−0.73 to −0.41
SAB	0.16	0.24	0.68	0.495	−0.31 to 0.64
Mean /s/ spectral centroid	1.79	0.39	4.62	<0.001	1.03 to 2.55
Time point*SAB	0.25	0.08	3.04	0.002	0.09 to 0.41
Time point* Mean /s/ spectral centroid	1.02	0.14	7.16	<0.001	0.74 to 1.31
SAB* Mean /s/ spectral centroid	0.21	0.37	0.57	0.57	−0.52 to 0.94
Time point*SAB* Mean /s/ spectral centroid	−0.43	0.14	−3.01	0.003	−0.71 to −0.15

## 4. Discussion

In this study, we examined the development of gender differences in speech by examining four acoustic measures of the speech of 55 AMAB and 55 AFAB children longitudinally: aVTL, VSD, f0, and /s/ spectral centroid. We also examined which of these acoustic variables allow adults to discern children’s SAB. We first asked how AMAB and AFAB children differed for each of the four acoustic measures. Our correlation analysis of the four acoustic variables showed a considerably weak relationship between the four acoustic variables. Those speech features, which index gender in adults, differed in how they were manifested by gender and age. One hypothesis suggested by this finding is that children deploy these features differently as they develop knowledge of the specific ways that each of these features indexes gender in adults. This hypothesis could be evaluated with studies that include measures of both children’s production of these variables and their use of them to evaluate the gender of others’ speech.

An alternative hypothesis is that the development of these features is driven by development in speech motor control. While this hypothesis cannot be tested directly with these data, there is evidence against this explanation in our data. We found that AMAB and AFAB children produced /s/ differently at 3 years of age: AMAB children produced /s/ with lower spectral centroid (i.e., a less fronted /s/) than AFAB children, mirroring the pattern in adults. Normative studies of speech development have found /s/ to be later acquired (Smit et al., 1990), which Kent (1992) attributed to the complex tongue-shapes required to create the narrow channel in the /s/ constriction that generates turbulent airflow. The fact that children’s production of /s/ reflected gender by 3 years of age suggests that developmental changes in the parameters that code gender are not due entirely – or perhaps even primarily – to motor control.

The current findings contrast with the findings by Li (2017), who studied the gender differences of sibilants /s/, /ʂ/, and /ɕ/ in children acquiring Mandarin as a first language. In the regional variety of Mandarin that Li studied, adult women and men differ strongly in their production of /ɕ/, with women producing an especially high centroid frequency. Li’s results showed no gender differences in fricative acoustics at 4 years of age. In contrast, gender differences in /ɕ/ mirroring those of adult men and women emerged at 6 years of age, which led to the hypothesis that social-indexical knowledge of speech is learned after the mastery of phonemes. However, our results indicate that children produced the gendered patterns of /s/ as early as 2.5 years of age, which suggests that the acquisition of gender marking occurs considerably earlier than Li’s data would lead us to predict.

In addition, we also found evidence of a longer aVTL in AMAB than the AFAB children at 5 years of age. There was, however, no evidence of gender differences in VSD and f0. These findings showed that gendered patterns of speech are learned relatively early. Previous research has not found differences in vocal-tract size and shape in children this young (Vorperian et al., 2009). Hence, while we do not have direct measures of vocal-tract size and length for these children, we regard it as unlikely that the aVTL differences reflect anatomical differences between children and instead reflect the manipulation of aVTL to convey their emerging gender. While it is not our intention in this paper to extrapolate these results to an anatomical dimension, we do point out that the model result suggests that there was only a 0.1 cm difference in aVTL between AMAB and AFAB children. Nonetheless, our finding is the first study to date to show gender differences in aVTL in children of 5 years of age. Previous studies from Cartei et al. (2014) were able to find evidence of gender difference in aVTL in older groups of children (aged 8–9). The specific reason that may lead to gender differences in children’s aVTL is unclear from the current data. One possibility is that some children are manipulating their aVTL by lowering or raising the larynx, by rounding or retracting the lips, or by a combination of these two manoeuvres across the production of all vowels. Another possibility is that some children are producing vowel-specific gendered phonetic features. For example, a group of children may be fronting their /u/ vowels more than the other group, which ultimately leads to differences in aVTL. As we have no hypothesis for vowel-specific features in the current study, future research is required to disentangle these issues. Even if this is ultimately found to be the case, we find it revealing that the vowel-specific manipulations enhance the differences in actual VTL between adult men and women.

Another important contribution of these acoustic findings is that children’s expression of gender through speech is not just a mere reflection of adult patterns; instead, children appear to use speech features that index gender selectively. However, it is unclear whether children are perceptually sensitive to some variables or if they are able to express only some of the gender cues through articulation. Both f0 and aVTL are arguably the most salient sex-dimorphic features in cisgender men and women, yet we found no evidence of gender difference in f0. Furthermore, we may question why gender differences are not present in the current data set if it is such a salient gendered speech feature. Particularly, evidence from a previous study showed that older groups of children were capable of manipulating f0 when asked to perform a masculine or feminine character (Cartei, Garnham, et al., 2019). It may be possible that children are aware of gender differences in f0, but they are not actively using f0 in expressing the gender of their own SAB. In other words, children manipulate f0 only when expressing other genders, but not in their daily speech. Alternatively, the null effect of f0 differences prior to 5 years of age may simply indicate that young children lack the fine motor control capability to manipulate f0. Previous studies on children’s production of voicing contrast showed that children are incapable of producing consistent covert contrast prior to 5 years of age (Hitchcock, 2005), which suggests a lack of fine motor control of glottal opening or overall vocal-fold function. Such evidence on articulatory limitations may explain why children do not manipulate their f0s as gendered cues of speaking. Finally, it is possible that the null effect of gender difference in children’s f0 is a consequence of this investigation’s focus on single words. Children may use f0 variation at the prosodic level to convey gender in connected speech, but not in single words. That hypothesis could be tested in a future study by comparing the ways that children convey gender in different types of speech samples.

Overall, our findings of gender differences in children’s speech lead us to the hypothesis that children are aware of the socially meaningful nature of variation in /s/ and aVTL at the earliest stage of language acquisition. The /s/ sound is a robust phonetic variable that differs acoustically between cisgender men and women and is highly associated with perceived masculinity and femininity (Munson, 2007). aVTL is a robust sex-dimorphic feature in adult speech. Our data may suggest that speech sound learning in children is beyond the phonemic level, as it involves the concurrent learning of social-indexical knowledge. This hypothesis is in contrast to most theories of language acquisition, which de-emphasize the acquisition of social-indexical knowledge, as reviewed recently by Johnson and White (2020). Further research is required to investigate the cognitive mechanisms of acquiring social-indexical knowledge of speech in children.

The final research question concerns what acoustic cues predict the perceived gender ratings obtained from adult listeners. We found that aVTL, VSD, and mean f0 all contributed to the adults’ perception of children’s gender. Among these acoustic features, aVTL is the strongest predictor of gender ratings, as it has the largest absolute coefficient when compared with mean f0 and VSD. Children’s aVTL also showed a consistent effect on their perceived gender ratings across both TPs and SAB. Our finding is supported by the results of a perceptual experiment done by Cartei and Reby (2013), in which the authors manipulated formant frequency spacing (ΔF) of the speech of 8-year-olds. It was found that masculinity ratings increased as ΔF decreased. Moreover, Munson et al. (2022) argued that the differences in perceived gender ratings were primarily attributed to AMAB children being rated as more “boy-like” at 5 years of age compared to 3 years. In the current study, we found supporting acoustic evidence that the gender differences in aVTL were present in the 5-year-olds, with the difference driven by the increase in aVTL in AMAB children from 3 to 5 years of age. Taken together, these findings suggest that aVTL is a robust cue of gender to adult listeners. Yet, whether listeners are sensitive to vowel-specific features requires further investigation.

While we found both VSD and f0 predict the ratings of children’s perceived gender, we did not find gender differences in VSD or f0 in children of 3 or 5 years of age. A similar effect of f0 on perceived gender ratings was also attested in recent studies with German-speaking children (Funk et al., 2023; Simpson et al., 2017). In that study, the authors found no f0 differences between AMAB and AFAB children of 6 to 8 years of age. Yet, f0 was the only acoustic parameter that predicted the perceived gender ratings of German-speaking children. Together, these results suggest that listeners’ perception of gendered speech reflects their expectation or *stereotyped* knowledge of gendered speech: they may believe that larger vowel spaces and higher f0s are associated with female talkers. Our results strengthen the constructionist view that gender is applicable to speech perception. Future research should examine how the ratings of perceived gender are influenced by listeners’ unique understandings of gender, as described in Tripp and Munson (2022).

In addition, we re-analysed the prediction of children’s perceived gender ratings using mean /s/ spectral centroid from a much larger set of /s/ tokens from Munson et al. (2022). The current analysis showed a consistent effect of /s/ acoustics on gender ratings and that the effect appears to be stronger in the 5-year-olds. This may be due to /s/ being a later-acquired consonant. A previous study by Barreda and Assmann (2021) demonstrated that the estimation of a talker’s age and gender was integrated in the speech perception process. In the current study, listeners may have been aware of children’s age and hence paid less attention to /s/ acoustics when rating children’s gender. Nonetheless, the fact that a single sound of /s/ is sufficient to cue the gender of the 5 year-olds contrasts with the findings of previous studies by Barreda and Assmann (2021) and Simpson et al. (2017), who concluded that gender was apparent only in prosodic features and not in segmental ones.

To conclude, our study provided the acoustic evidence that children of 3 to 5 years of age mark gender through variation in vowel and consonant features. Our findings that children’s production of single words encodes gender highlight that the acquisition of social-indexical knowledge is integrated with and fundamental to language learning.

## Supporting information

Wong et al. supplementary material Wong et al. supplementary material

## Supplementary material

The supplementary material for this article can be found at http://doi.org/10.1017/S030500092500011X.

## Long descriptions

### Table 1. Long description

The table is divided into two main horizontal sections: First time point and Last time point. Each section contains columns organized by I P A vowel symbols with their respective sample sizes (n).

First time point section:

* /i/ (n = 293): sheep.

* /ɪ/ (n = 1366): dinner, dish, kitchen, kitty, scissors, tickle, give, sick.

* /ɛ/ (n = 831): share, teddy bear, get.

* /æ/ (n = 1019): candy, cat, daddy, dance, sandwich, sad.

* /u/ (n = 666): tooth, shoe, soup.

* /ʊ/ (n = 318): cookie, good.

* /ʌ/ (n = 1204): sun, cup, shovel, tongue, tummy, duck, gum.

* /ɑ/ (n = 1039): car, dog, door, garbage, sock.

* /oʊ/ (n = 133): soap.

Last time point section:

* /i/ (n = 687): cheese, keys, reading, teacher, queen, sheep, wheel.

* /ɪ/ (n = 1053): cereal, chicken, kitchen, kitten, scissors, sink, sister, tickle, window, ship.

* /ɛ/ (n = 1218): chair, red, seven, sharing, teddy bear, twelve, twenty, web, shell, tent, wet.

* /æ/ (n = 1167): candle, candy, cracker, rabbit, sandbox, sandwich, shadow, cat, trash.

* /u/ (n = 737): roof, room, shoes, suitcase, toothbrush, soup.

* /ʊ/ (n = 605): cookie, sugar, wolf, woman, wood.

* /ʌ/ (n = 1539): cousin, cutting, running, summer, tongue, trouble, tummy, sun, cup, shovel, truck.

* /ɑ/ (n = 710): coffee, crawl, walk, washcloth, washer, water, rock.

* /aɪ/ (n = 86): sidewalk.

Phonetic transcriptions are provided for every word. Asterisks and italics indicate specific subsets used for gender rating or /s/ analysis.

Navigate back to Table 1..

### Figure 1. Long description

The scatter plot uses a coordinate system where the horizontal X axis represents Delta dash F Normalized F 2, with values decreasing from 2.0 on the left to 1.0 on the right. The vertical Y axis represents Delta dash F Normalized F 1, with values increasing from 0.3 at the top to 0.7 at the bottom. At the center of the diagram, located approximately at X equals 1.4 and Y equals 0.53, is a solid black dot labeled in the legend as Center of Vowel Space. Numerous gray arrows radiate outward from this central dot toward individual vowel tokens represented by International Phonetic Alphabet symbols. The vectors extend in all directions. To the top left, arrows point toward lowercase i and small capital I symbols. To the top right, arrows point toward lowercase u and upsilon symbols. To the bottom left, arrows point toward epsilon and ash symbols. To the bottom right, arrows point toward script a, turned v, and open o symbols. The length of each arrow represents the Euclidean distance from the center to that specific vowel token.

Navigate back to Figure 1..

### Table 2. Long description

The table consists of four columns and three primary data rows representing acoustic variables. The columns are labeled a V T L, V S D, Mean f 0, and Mean forward slash s forward slash.

* Row 1, V S D: The correlation with a V T L is 0.24 with a confidence interval of 0.05 to 0.41.

* Row 2, Mean f 0: The correlation with a V T L is 0.04 with a confidence interval of minus 0.15 to 0.23. The correlation with V S D is 0.12 with a confidence interval of minus 0.07 to 0.30.

* Row 3, Mean forward slash s forward slash: The correlation with a V T L is minus 0.29 with a confidence interval of minus 0.46 to minus 0.10. This value is marked with an asterisk indicating p is less than .05. The correlation with V S D is 0.18 with a confidence interval of minus 0.02 to 0.36. The correlation with Mean f 0 is 0.15 with a confidence interval of minus 0.05 to 0.34.

Diagonal cells representing the correlation of a variable with itself are marked with dashes.

Navigate back to Table 2..

### Table 3. Long description

The table consists of four columns for the variables a V T L, V S D, Mean f 0, and Mean /s/.

* Row 1, V S D: The correlation with a V T L is 0.16 with a confidence interval of negative 0.03 to 0.33.

* Row 2, Mean f 0: The correlation with a V T L is negative 0.06 with a confidence interval of negative 0.24 to 0.13. The correlation with V S D is 0.25 with a confidence interval of 0.07 to 0.42, marked with a single asterisk indicating p is less than .05.

* Row 3, Mean /s/: The correlation with a V T L is negative 0.39 with a confidence interval of negative 0.54 to negative 0.22, marked with three asterisks indicating p is less than .001. The correlation with V S D is 0.003 with a confidence interval of negative 0.19 to 0.19. The correlation with Mean f 0 is 0.23 with a confidence interval of 0.05 to 0.40.

Diagonal cells representing self-correlation are marked with dashes.

Navigate back to Table 3..

### Table 4. Long description

The table contains six columns: Variable, Estimate, S E, z, p, and 95% C I.

* Intercept: Estimate 11.57, S E 0.03, z 342.41, p less than 0.001, 95% C I 11.50 to 11.64.

* Time point (F T P equals negative 1, L T P equals 1): Estimate 0.18, S E 0.02, z 7.79, p less than 0.001, 95% C I 0.13 to 0.22.

* S A B (A M A B equals negative 1, A F A B equals 1): Estimate negative 0.09, S E 0.03, z negative 2.54, p 0.013, 95% C I negative 0.15 to negative 0.02.

* Time point asterisk S A B: Estimate negative 0.10, S E 0.02, z negative 4.46, p less than 0.001, 95% C I negative 0.15 to negative 0.06.

Navigate back to Table 4..

### Figure 2. Long description

The vertical y-axis represents Acoustic vocal-tract length in c m, ranging from 10.5 to 12.5. The horizontal x-axis categorizes data by Sex assigned at birth, labeled A M A B and A F A B.

Panel 1, titled First time-point F T P, shows two violin plots. The yellow A M A B plot has a median near 11.4 c m with a box plot spanning roughly 11.1 to 11.7 c m. The purple A F A B plot has a slightly higher median near 11.5 c m with a similar box plot range.

Panel 2, titled Last time-point L T P, shows an upward trend for both groups. The yellow A M A B plot now has a median near 11.9 c m, with the distribution extending up to nearly 13.0 c m. The purple A F A B plot shows a median near 11.6 c m, with a distribution spanning from 10.6 to 12.6 c m.

In both panels, the violin shapes indicate density, with the widest sections corresponding to the most frequent measurements around the medians.

Navigate back to Figure 2..

### Table 5. Long description

The table consists of six columns: Predictor, Estimate, S E, z, p, and 95% C I.

* Intercept: Estimate 0.40, S E 0.02, z 21.16, p less than 0.001, 95% C I 0.36 to 0.43.

* Time point (F T P equals negative 1, L T P equals 1): Estimate 0, S E 0, z 1.26, p 0.21, 95% C I 0 to 0.01.

* S A B (A M A B equals negative 1, A F A B equals 1): Estimate 0, S E 0, z 0.43, p 0.67, 95% C I 0 to 0.01.

* Mean vowel duration: Estimate 0.02, S E 0.09, z 0.24, p 0.81, 95% C I negative 0.16 to 0.20.

Navigate back to Table 5..

### Figure 3. Long description

The two panels are titled First time-point F T P and Last time-point L T P. The vertical Y-axis represents Vowel space dispersion with values from 0.3 to 0.5. The horizontal X-axis is labeled Sex assigned at birth with categories A M A B and A F A B.

In the F T P panel on the left

* The A M A B group is represented by a yellow violin plot. It shows a wide distribution centered around 0.4 with a long tail extending down to approximately 0.25. The internal white box plot indicates a median just below 0.4.

* The A F A B group is represented by a purple violin plot. It is wider at the top than the A M A B group, with a median slightly above 0.4 and a tail extending down to 0.28.

In the L T P panel on the right

* The A M A B group yellow violin plot has shifted upward, with the bulk of the distribution between 0.35 and 0.45 and a median above 0.4. The lower tail is shorter, ending near 0.3.

* The A F A B group purple violin plot appears more compressed vertically compared to its F T P counterpart. The distribution is concentrated between 0.35 and 0.45 with a median at 0.4 and a much shorter lower tail.

Navigate back to Figure 3..

### Table 6. Long description

The table consists of six columns: Predictor, Estimate, S E, z, p, and 95% C I.

* Intercept: Estimate 289.91, S E 3.09, z 93.81, p less than 0.001, 95% C I 283.82 to 296.01.

* Time point (F T P equals negative 1, L T P equals 1): Estimate negative 14.86, S E 2.15, z negative 6.91, p less than 0.001, 95% C I negative 19.12 to negative 10.61.

* S A B (A M A B equals negative 1, A F A B equals 1): Estimate 1.54, S E 2.41, z 0.64, p 0.53, 95% C I negative 3.24 to 6.31.

Navigate back to Table 6..

### Figure 4. Long description

The chart consists of two side-by-side panels. The y-axis is labeled F 0 in H z and ranges from 100 to 500. The x-axis is labeled Sex assigned at birth with categories A M A B and A F A B.

Left Panel: First time-point F T P.

* A M A B: A yellow violin plot with a central box plot. The median is approximately 300 H z. The distribution is concentrated between 250 and 350 H z with tails extending from 130 to 480 H z.

* A F A B: A purple violin plot with a central box plot. The median is approximately 300 H z. The distribution is similar to A M A B with tails from 130 to 490 H z.

Right Panel: Last time-point L T P.

* A M A B: A yellow violin plot. The median has dropped to approximately 260 H z. The bulk of the distribution is lower, between 230 and 300 H z, with tails from 100 to 450 H z.

* A F A B: A purple violin plot. The median is approximately 275 H z. The distribution is slightly higher than the A M A B group in this time point, with tails from 100 to 450 H z.

Navigate back to Figure 4..

### Table 7. Long description

The table consists of six columns: Predictor, Estimate, S E, z, p, and 95% C I.

* Intercept: Estimate 6731.06, S E 79.26, z 84.92, p less than 0.001, 95% C I 6573.75 to 6888.37.

* Time point (F T P equals minus 1, L T P equals 1): Estimate 232.84, S E 53.10, z 4.39, p less than 0.001, 95% C I 127.66 to 338.03.

* S A B (A M A B equals minus 1, A F A B equals 1): Estimate 210.18, S E 71.93, z 2.92, p 0.004, 95% C I 67.34 to 353.01.

Navigate back to Table 7..

### Figure 5. Long description

The two panels are arranged horizontally. The y-axis is labeled Spectral centroid of forward slash s forward slash in k H z with a scale from 4 to 10. The x-axis is labeled Sex assigned at birth with categories A M A B and A F A B. Each violin plot contains an internal box plot.

Left Panel: First time-point F T P.

* The A M A B violin is yellow. The median is approximately 6.6 k H z. The interquartile range spans from roughly 5.5 to 7.6 k H z. The distribution is elongated with a peak near 9.6 k H z and a base near 3.4 k H z.

* The A F A B violin is purple. The median is higher at approximately 7.0 k H z. The interquartile range spans from roughly 6.2 to 7.8 k H z. The distribution is wider in the middle than the A M A B group.

Right Panel: Last time-point L T P.

* The A M A B violin is yellow. The median is approximately 6.9 k H z. The interquartile range spans from roughly 6.1 to 7.7 k H z. The distribution shows a slight upward shift compared to F T P.

* The A F A B violin is purple. The median is the highest of all groups at approximately 7.5 k H z. The interquartile range spans from roughly 6.5 to 8.1 k H z. The distribution is concentrated higher on the y-axis than the other three groups.

Navigate back to Figure 5..

### Table 8. Long description

The table contains 10 rows of statistical data with columns for Estimate, S E, z, p-value, and 95% C I.

* Intercept: Estimate 0.33, S E 0.16, z 2.04, p 0.041, C I 0.01 to 0.64.

* Time point (F T P equals negative 1, L T P equals 1): Estimate negative 0.06, S E 0.05, z negative 1.12, p 0.262, C I negative 0.16 to 0.04.

* S A B (A M A B equals negative 1, A F A B equals 1): Estimate negative 0.08, S E 0.07, z negative 1.13, p 0.258, C I negative 0.22 to 0.06.

* Mean f 0: Estimate 0.15, S E 0.13, z 1.12, p 0.264, C I negative 0.11 to 0.4.

* a V T L: Estimate negative 1.15, S E 0.21, z negative 5.51, p less than 0.001, C I negative 1.56 to negative 0.74.

* V S D: Estimate 0.45, S E 0.23, z 1.98, p 0.047, C I 0.01 to 0.89.

* Time point asterisk S A B: Estimate 0.19, S E 0.05, z 3.52, p less than 0.001, C I 0.08 to 0.29.

* S A B asterisk Mean f 0: Estimate 0.41, S E 0.13, z 3.13, p 0.002, C I 0.16 to 0.67.

* Time point asterisk Mean f 0: Estimate 0.06, S E 0.10, z 0.61, p 0.545, C I negative 0.13 to 0.25.

* Time point asterisk S A B asterisk Mean f 0: Estimate negative 0.24, S E 0.10, z negative 2.56, p 0.011, C I negative 0.43 to negative 0.06.

Navigate back to Table 8..

### Figure 6. Long description

The graph consists of two side-by-side panels. The y-axis is labeled Perceived gender ratings of children's speech, ranging from 0 ‘Definitely a boy’ to 1 ‘Definitely a girl’. The x-axis is labeled Acoustic vocal-tract length in c m, ranging from 11 to 13. A legend to the right identifies two time-points: F T P represented by a solid line and L T P represented by a dashed line.

* Left Panel (A M A B): Shows a negative linear correlation. At 11 c m, ratings are between 0.5 and 0.75. As vocal-tract length increases to 13 c m, ratings for both F T P and L T P decrease to approximately 0.3. The L T P dashed line starts higher than the F T P solid line but converges at the end.

* Right Panel (A F A B): Also shows a negative linear correlation. At 11 c m, ratings are higher than the A M A B group, starting near 0.75. As vocal-tract length increases to 13 c m, ratings decrease to approximately 0.4. Similar to the first panel, the L T P dashed line has a steeper negative slope than the F T P solid line.

Both panels include shaded error bands around the regression lines and a background of faint vertical scatter points.

Navigate back to Figure 6..

### Figure 7. Long description

The graph consists of two side-by-side panels. The Y-axis is labeled Perceived gender ratings of children's speech, ranging from 0 ‘Definitely a boy’ to 1 ‘Definitely a girl’. The X-axis is labeled Vowel space dispersion Mean Euclidean distance from center of vowel space, ranging from 0.3 to 0.5. A legend on the right identifies two time-points: F T P represented by a solid line and L T P represented by a dashed line.

* Left Panel: A M A B. The F T P solid yellow line remains nearly flat at a rating of 0.5 across the X-axis. The L T P dashed yellow line shows a slight positive linear increase, starting near 0.4 at 0.25 dispersion and rising toward 0.5 at 0.5 dispersion.

* Right Panel: A F A B. The F T P solid maroon line is relatively flat, hovering just above 0.5. The L T P dashed maroon line shows a positive linear trend, starting below the F T P line at 0.5 and rising to approximately 0.65 as dispersion increases to 0.5.

Both panels include shaded gray areas around the lines representing confidence intervals and a background of faint vertical scatter points.

Navigate back to Figure 7..

### Figure 8. Long description

The figure consists of two side-by-side panels. The y-axis is labeled Perceived gender ratings of children's speech, ranging from 0 ‘Definitely a boy’ to 1 ‘Definitely a girl’. The x-axis is labeled Mean f 0 in H z, ranging from 200 to 350.

Left Panel: Titled A M A B. This panel shows two yellow regression lines with gray confidence intervals. The solid line representing F T P remains nearly flat at a rating of 0.5 across the frequency range. The dashed line representing L T P is slightly lower, starting around 0.43 and showing a very slight upward slope toward 0.45.

Right Panel: Titled A F A B. This panel shows two purple regression lines with gray confidence intervals. Both lines show a positive linear increase. The solid line for F T P starts at 0.5 and rises to approximately 0.65. The dashed line for L T P starts slightly higher at 0.5 and rises more steeply to approximately 0.7.

Legend: Located to the right of the panels, titled Time-point. A solid line corresponds to F T P and a dashed line corresponds to L T P. Background data points are visible as faint vertical clusters in both panels.

Navigate back to Figure 8..

### Table 9. Long description

The table contains six columns: Variable, Estimate, S E, z, p, and 95% C I.

* Intercept: Estimate -0.96, S E 0.25, z -3.82, p less than 0.001, C I -1.45 to -0.47.

* Time point: Estimate -0.57, S E 0.08, z -6.88, p less than 0.001, C I -0.73 to -0.41.

* S A B: Estimate 0.16, S E 0.24, z 0.68, p 0.495, C I -0.31 to 0.64.

* Mean forward slash s forward slash spectral centroid: Estimate 1.79, S E 0.39, z 4.62, p less than 0.001, C I 1.03 to 2.55.

* Time point asterisk S A B: Estimate 0.25, S E 0.08, z 3.04, p 0.002, C I 0.09 to 0.41.

* Time point asterisk Mean forward slash s forward slash spectral centroid: Estimate 1.02, S E 0.14, z 7.16, p less than 0.001, C I 0.74 to 1.31.

* S A B asterisk Mean forward slash s forward slash spectral centroid: Estimate 0.21, S E 0.37, z 0.57, p 0.57, C I -0.52 to 0.94.

* Time point asterisk S A B asterisk Mean forward slash s forward slash spectral centroid: Estimate -0.43, S E 0.14, z -3.01, p 0.003, C I -0.71 to -0.15.

Navigate back to Table 9..

### Figure 9. Long description

The graph consists of two horizontal panels. The y-axis is labeled Perceived gender ratings of children's speech, ranging from 0 ‘Definitely a boy’ to 1 ‘Definitely a girl’. The x-axis is labeled Spectral centroid of forward slash s forward slash in k H z, ranging from 4 to 9. A legend on the right identifies two time-points: F T P represented by a solid line and L T P represented by a dashed line.

* Left Panel: A M A B. Individual data points are scattered in light yellow. The F T P solid yellow line shows a slight negative linear trend, starting around 0.5 at 4 k H z and ending just below 0.5 at 9 k H z. The L T P dashed yellow line shows a sharp positive linear increase, starting near 0.1 at 4 k H z and rising to approximately 0.7 at 9 k H z. The lines intersect at roughly 6.5 k H z.

* Right Panel: A F A B. Individual data points are scattered in light purple. The F T P solid purple line is nearly horizontal, maintaining a rating slightly above 0.5 across the entire x-axis. The L T P dashed purple line shows a strong positive linear trend, starting near 0.4 at 4 k H z and rising to approximately 0.8 at 9 k H z. The lines intersect at roughly 6.5 k H z.

Both panels include grey shaded areas around the lines representing confidence intervals.

Navigate back to Figure 9..
