# Supplementary material for: Gendered speech development in early childhood: Evidence from a longitudinal study of vowel and consonant acoustics
Source: J Child Lang. 2025 Apr 4;53(4):759–86. doi: 10.1017/S030500092500011X (PMC13320588; doi:10.1017/S030500092500011X)

Scatterplots on the correlation between aVTL, VSD, mean f0 and /s/ spectral centroid in first time-point (FTP) and last time-point (LTP).

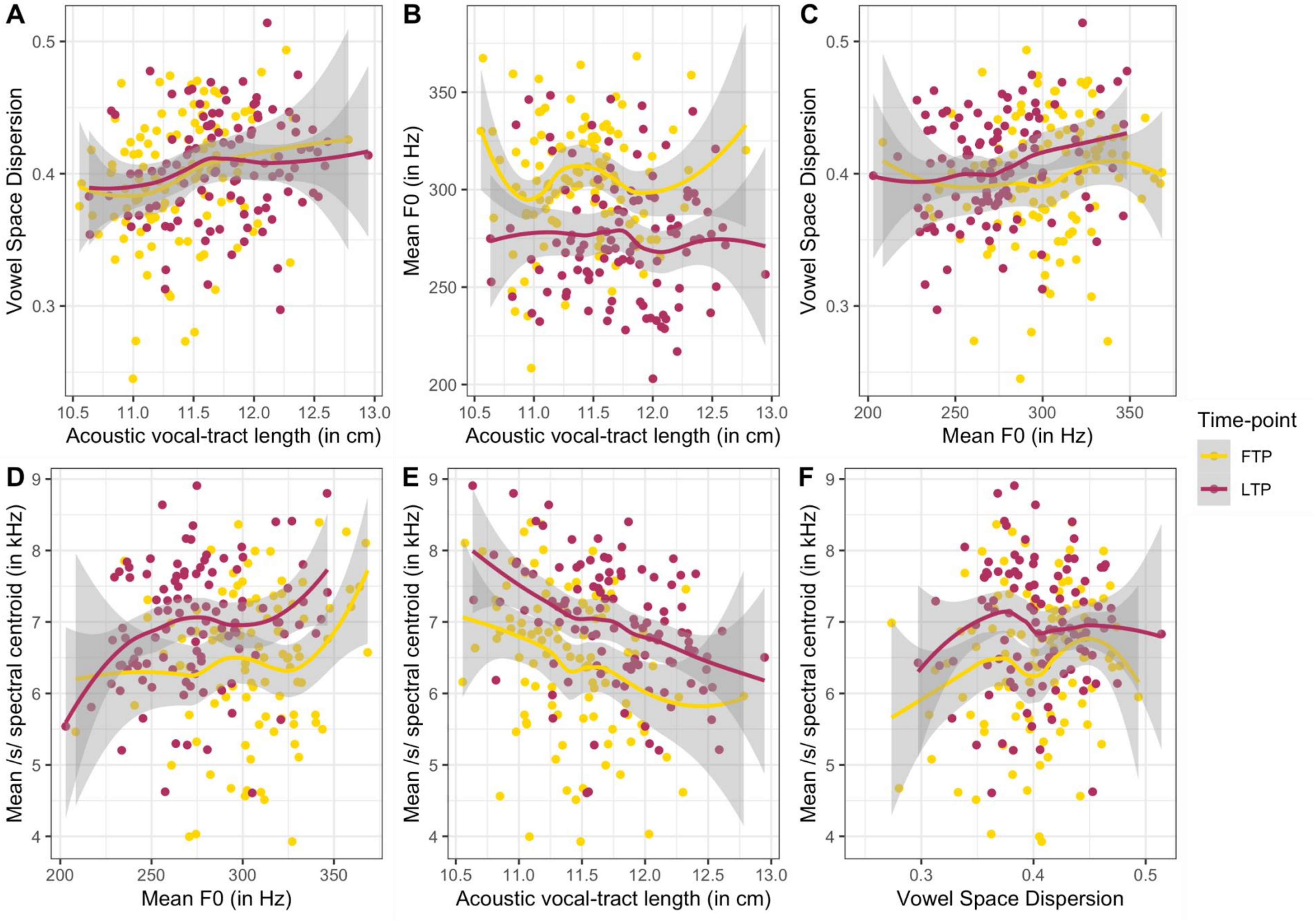

Supplement: Wong et al. supplementary material [file S030500092500011Xsup001.pdf]
